# A Novel Patient-Tailored, Cumulative Neurotechnology-Based Therapy for Upper-Limb Rehabilitation in Severely Impaired Chronic Stroke Patients: The *AVANCER* Study Protocol

**DOI:** 10.3389/fneur.2022.919511

**Published:** 2022-07-07

**Authors:** Claudia Bigoni, Sarah B. Zandvliet, Elena Beanato, Andrea Crema, Martina Coscia, Arnau Espinosa, Tina Henneken, Julie Hervé, Meltem Oflar, Giorgia G. Evangelista, Takuya Morishita, Maximilian J. Wessel, Christoph Bonvin, Jean-Luc Turlan, Niels Birbaumer, Friedhelm C. Hummel

**Affiliations:** ^1^Defitech Chair of Clinical Neuroengineering, Center for Neuroprosthetics (CNP) and Brain Mind Institute (BMI), École Polytechnique Fédérale de Lausanne (EPFL), Geneva, Switzerland; ^2^Defitech Chair of Clinical Neuroengineering, Center for Neuroprosthetics (CNP) and Brain Mind Institute (BMI), École Polytechnique Fédérale de Lausanne (EPFL), Clinique Romande de Réadaptation, Sion, Switzerland; ^3^Department of Rehabilitation, Donders Institute for Brain, Cognition and Behaviour, Radboud University Medical Centre, Nijmegen, Netherlands; ^4^Clinical Neuroscience, University of Geneva Medical School, Geneva, Switzerland; ^5^Bertarelli Foundation Chair in Translational Neuroengineering, Centre for Neuroprosthetics and Institute of Bioengineering, School of Engineering, École Polytechnique Fédérale de Lausanne (EPFL), Lausanne, Switzerland; ^6^Wyss Center for Bio and Neuroengineering, Geneva, Switzerland; ^7^confinis AG, Sursee, Switzerland; ^8^Division of Neurology, Wallis Hospital, Sion, Switzerland; ^9^Department of Neurological Rehabilitation, Clinique Romande de Réadaptation Suva, Sion, Switzerland; ^10^Department of Medical Psychology and Behavioral Neurobiology, University of Tübingen, Tübingen, Germany

**Keywords:** stroke, rehabilitation, brain computer interface, personalized, tDCS, upper limb

## Abstract

Effective, patient-tailored rehabilitation to restore upper-limb motor function in severely impaired stroke patients is still missing. If suitably combined and administered in a personalized fashion, neurotechnologies offer a large potential to assist rehabilitative therapies to enhance individual treatment effects. AVANCER (clinicaltrials.gov NCT04448483) is a two-center proof-of-concept trial with an individual based cumulative longitudinal intervention design aiming at reducing upper-limb motor impairment in severely affected stroke patients with the help of multiple neurotechnologies. AVANCER will determine feasibility, safety, and effectivity of this innovative intervention. Thirty chronic stroke patients with a Fugl-Meyer assessment of the upper limb (FM-UE) <20 will be recruited at two centers. All patients will undergo the cumulative personalized intervention within two phases: the first uses an EEG-based brain-computer interface to trigger a variety of patient-tailored movements supported by multi-channel functional electrical stimulation in combination with a hand exoskeleton. This phase will be continued until patients do not improve anymore according to a quantitative threshold based on the FM-UE. The second interventional phase will add non-invasive brain stimulation by means of anodal transcranial direct current stimulation to the motor cortex to the initial approach. Each phase will last for a minimum of 11 sessions. Clinical and multimodal assessments are longitudinally acquired, before the first interventional phase, at the switch to the second interventional phase and at the end of the second interventional phase. The primary outcome measure is the 66-point FM-UE, a significant improvement of at least four points is hypothesized and considered clinically relevant. Several clinical and system neuroscience secondary outcome measures are additionally evaluated. AVANCER aims to provide evidence for a safe, effective, personalized, adjuvant treatment for patients with severe upper-extremity impairment for whom to date there is no efficient treatment available.

## Introduction

Stroke-related disabilities are highly heterogeneous, with frequent occurrence of motor deficits, affecting around 80% of stroke survivors (1). The severity of impairment is diverse, with around 25% of patients showing little to no motor recovery of the upper-limb (2, 3). There is now a bouquet of different types of treatments for upper-limb motor rehabilitation (4–7) ranging from mirror and constrained-induced movement therapy to neurotechnology-based approaches, such as robotics, non-invasive brain stimulation, peripheral nerve stimulation or virtual reality (8, 9). However, most of these therapies may only be applied successfully to subgroups of patients; in particular, treatment strategies for severely impaired patients are limited and non-effective. Specifically, this population may benefit from neurotechnology-based interventions as they will allow these patients personalized, high-intensity and repetitive training (9). Neurotechnologies for stroke motor rehabilitation comprise robot- and exoskeleton-, functional electrical stimulation (FES), brain stimulation- and brain computer interface (BCI)-based interventions. We refer the reader to (5, 8–15) for detailed reviews on the use of these technologies in motor rehabilitation for stroke patients. In addition to the above-mentioned characteristics, these assistive devices are modular, allowing their use singularly or in combination with each other. With the second approach, better results might be achieved due to the potential of synergistic, additive/supra-additive effects of complementary features and interventional targets (8). Robot- and FES-based strategies target the peripheral nervous system by activating muscles, inducing movements and providing sensory feedback. In addition, FES allows for activation of the efferent pathways if used with supra motor threshold amplitudes (16). Brain stimulation and BCI target the central nervous system. The former attempts to modulate the underlying excitability and achieve neuro-plastic effects crucial for learning and reorganization (10, 17, 18); the second one aims to enhance the engagement of the task-specific brain regions to perform the impaired functional tasks. Moreover, the BCI setup orchestrates the other neurotechnologies to reinstate a coherent interaction of efferent and afferent activity essential for behavioral improvement by decoding the recorded brain activity associated with the attempt to move the paretic upper extremity. The peculiarity of BCI is that it allows for functional efferent-afferent contingency, immediate feedback and reinforcement even without any movement capacity (19–21). Albeit promising, BCI as a therapy for upper-limb rehabilitation is still at its infancy and most of the randomized clinical trials (RCTs) are heterogeneous in terms of brain-directed machines used, movements performed, intervention duration and targeted population (13, 22–25). These variabilities are not specific to BCI therapies, but have also been reported in other stroke-related upper-limb rehabilitation treatments (13, 26). As highlighted by the Stroke Recovery and Rehabilitation Roundtables (SRRR) (27, 28) and a recent meta-analysis (29), trials controlled in terms of time point post-stroke and patient phenotype are still lacking. Precision medicine approaches are needed with the clear goal of creating treatment protocols able to adjust the therapy and its specific parameters to the condition and needs of the individual patient to maximize the effects. Meta-analyses have demonstrated a positive effect of dose-response in stroke motor-rehabilitation (30), yet choosing the correct dosage for each patient a-priori remains a challenge (27, 29, 31). Therefore, intervention duration should be individually adjusted along the therapy to allow every patient to achieve the maximum treatment response. Furthermore, the intervention itself may be dynamically adjusted by adding adjuvant treatments. This becomes of relevance when multiple neurotechnologies (or different approaches such as pharmacology) are combined. If each device has a precise target, they should be gradually combined in a patient-tailored fashion to maximize the results; for example starting from peripheral interventions with exoskeleton and FES to allow the training of upper extremity movement, toward a central, orchestrated engagement with the BCI-based approach, followed by further support of non-invasive brain stimulation for neuroplastic and reorganization processes in the large-scale network (32–34).

With the AVANCER (*Accident Vasculaire cérébrale et Apport des Neurotechnologies individualisées chez le patient sévère Chronique: une Etude clinique prospective visant à Restaurer la mobilité du membre supérieur*)^1^ proof-of-concept trial, we propose a personalized neurotechnology-based therapy for upper-limb motor rehabilitation in severe chronic stroke patients. To maximize treatment effects, we propose two main pillars. The first is the combination of neurotechnologies to leverage their synergistic effects. The second is the personalization in the intervention duration and in a therapy assisted by a hierarchical sequence of non-invasive neurotechnologies, i.e., BCI, FES, exoskeleton and transcranial direct current stimulation (tDCS). To acknowledge high-dimensional heterogeneity in the investigated population (e.g., lesion location and dose-response) and because we expect no natural recovery, a within subject design is the most appropriate and has been chosen to evaluate the following hypothesis: the proposed cumulative neurotechnologies-based intervention leads to an increase of at least 4 points on the Fugl-Meyer assessment for the upper extremity (FM-UE) (35) on average across the targeted population, at end of the sequential treatment.

The main objective of AVANCER is to maximize upper-limb motor improvement in severely impaired stroke patients by the end of the cumulative tailored treatment. Secondary aims are to investigate the rehabilitation process in terms of time, intervention, and patients' specifics; and to determine underlying neural and clinically relevant behavioral mechanisms by means of a multi-modal evaluation. Moreover, given the novelty of the protocol, we aim to study the feasibility and safety of this setup and design.

## Methods and Analysis

AVANCER is a sponsor-initiated, national multicenter (Campus Biotech, Geneva and Clinque Romande de Réadaptation, SUVA, Sion, Switzerland) proof-of-concept trial with an individual based cumulative longitudinal intervention design. All recruited patients undergo the intervention.

### Inclusion Criteria

**Figure 1:**
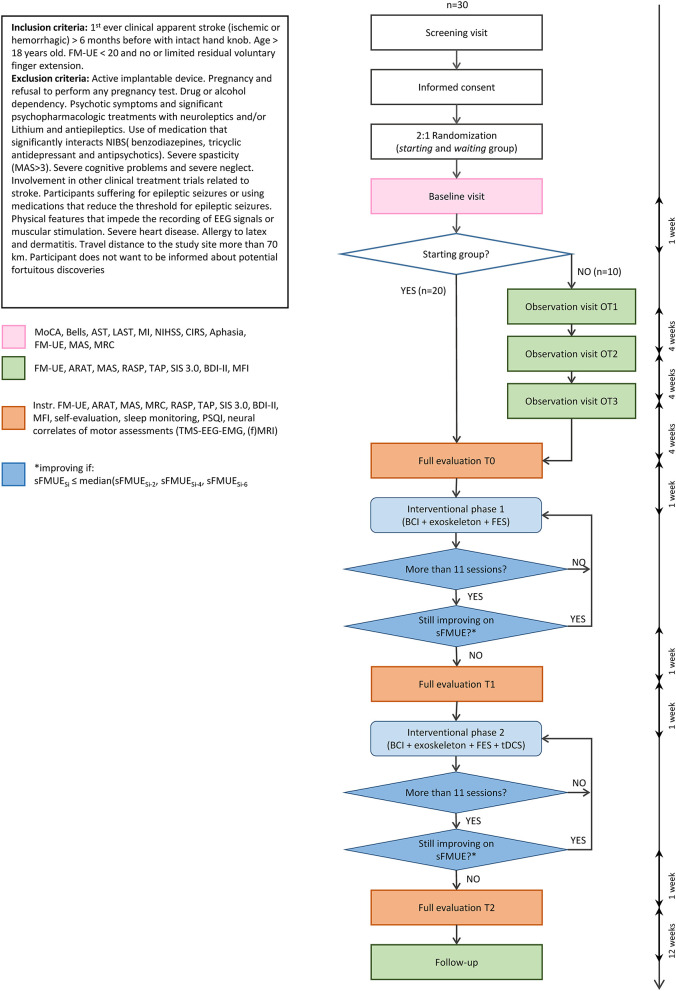
Flowchart of the AVANCER study with patient inclusion/exclusion criteria. The procedure flow shows the timeline with maximum delays between steps in terms of weeks. MoCA, Montreal Cognitive Assessment (36); Bells, Bells cancellation test (37); AST, apraxia screening of TULIA (38); LAST, language screening test for aphasia (39); MI, motricity index (40); NIHSS, NIH Stroke Scale (41); CIRS, Cumulative Illness Rating scale (42); FM-UE, Fugl-Meyer Assessment for the upper limb (35); MAS, modified Ashworth scale (43); MRC, Medical Research Council scale; ARAT, Action Research Arm Test (44); RASP, Rivermead Assessment of Somatosensory Performance (45); TAP, test of attentional performance (subsections of phasic alertness and divided attention) (46); SIS, Stroke Impact Scale 3.0 (47); BDI-II, Beck Depression Inventory scale (48); MFI, Multidimensional Fatigue Inventory (49); PSQI, Pittsburgh sleep quality index (50). sFM-UE is a short version of the FM-UE where the maximum is 54 and reflex items are removed.

For patients to be recruited in the study, they must fulfill the following requirements: be in a chronic stage and severely impaired. The former means that the stroke must have happened at least 6 months before enrollment; the second is defined by the FM-UE of below 20 points. Specifically, we include patients with limited residual voluntary finger extension measured with the FM-UE score of “hand mass extension” for which we accept a score below or equal to 1. Moreover, patients' MRIs are analyzed to assess that the hand-knob of the lesioned hemisphere is intact. The latter is evaluated case by case as follows: (1) if the stroke lesion does not affect the hand knob area, the hand knob is considered intact; (2) if the stroke lesion affects the hand knob, but the gray matter is not affected, the hand knob is considered intact; (3) if the gray matter of the hand knob area is affected, the hand knob is considered as non-intact and the patient is excluded. A patient is only eligible to participate if he/she understands the protocol and is able to consent. With each patient a cognitive screening with the Montreal Cognitive Assessment (MoCa) (36) is performed. Although there are no well-established cut-off scores due to limited reliability with patients with speech and languages problems, with a score of 18 or lower, upon consent with the patient, contact with the treating physician is made to verify if the patient is capable of understanding the study content. Full inclusion and exclusion criteria are reported in Figure 1.

### Recruitment

The two study sites (Geneva, Sion) are in continuous contact and close proximity to the respective hospitals (University Hospital of Geneva – HUG; Hôpital Valais de Sion - HVS). Moreover, the project has collaborations with three rehabilitation clinics in the areas (Clinique romande de réadaption – CRR; Berner Klink Montana, Crans-Montana; Clinique La Lignere, Gland). Recruitment is done through multiple channels such as patients' contact through hospitals, study presentation to patients by partnered clinics and leaflets at physicians' cabinets.

### Study Procedure

All thirty planned patients will receive a cumulative neurotechnologies-based intervention: (1) Interventional phase 1 (IP1) uses a BCI to control supra-motor threshold FES for the upper-limb and a hand exoskeleton for flexion and extension of the fingers with arm support; (2) Interventional phase 2 (IP2) adds tDCS to IP1.

The duration of both interventions is tailored for each patient and is determined according to improvement monitored through a short version of the FM-UE (sFM-UE) (35), consisting of 54 points excluding reflexes and coordination items. This short FM-UE is assessed every second session of intervention. The patient is considered to have no further prospect of recovery when no improvement in terms of sFM-UE is seen in the current session (Si), defined by:


(1) $$\begin{array}{r} {\text{sFMUE}}_{\text{Si}}\;\leq \text{ median}\left({\text{sFMUE}}_{\text{Si}-2},\;{\text{sFMUE}}_{\text{Si}-4},\;{\text{sFMUE}}_{\text{Si}-6}\right) \end{array}$$

When this condition is met, the intervention changes to a second stage and the same procedures take place to determine the end of *IP2*. A minimum of 11 sessions per intervention is provided and equation (1) is thus applied from the eleventh session of both phases. The session counting of *IP1* begins when the patient can govern the BCI (i.e., reaching 70% of accuracy in offline model validation); this choice is made to have a similar amount of *valid* therapy sessions in the two interventional phases. In addition to the therapies, every subject participates in three multimodal assessment visits: before *IP1* (T0), at the change to the second interventional phase (T1), and at the end of *IP2* (T2). A follow-up visit is scheduled 3 months after the end of *IP2*. The full procedure can be seen in Figure 1. Visits and interventions are conducted at the centers, in our laboratory spaces. Only screening and observation visits may be done at the patient's place of residence.

### Interventions

The heart of our interventional setup is a non-invasive electroencephalography (EEG)-based BCI that governs the activation of two actuators: a robotic glove and multi-channel FES.

#### Materials

Brain activity is recorded from a 16-channel electroencephalogram (16 Channel V-amp system, Brain Products GmbH, Gilching, Germany; Ag/AgCl electrodes on ActiCAP 10–20 system) covering the motor cortex of both hemispheres. The signals are sent to an external decoder that classifies if the incoming signal corresponds to a motor intention for the affected limb. When this condition is met, the two actuators (the exoskeleton and the FES) are triggered. The exoskeleton is a Gloreha Sinfonia (Idrogenet, Brescia, Italy) consisting of a cable-driven glove that can be used to flex and extend fingers (36). Thanks to the embedded stretch-sensors, we are able to monitor voluntary activation and perform assisted-as-needed movements. The proximal upper-limb can be activated by supra-motor threshold FES (Rehastim, Hasomed Germany with PALS electrodes, Axelgaard, Denmark) at seven different muscles: rhomboids, deltoid anterior and medialis, biceps brachialis, triceps, brachioradialis and extensor digitorum. Stimulation can be given to each muscle individually or in combination.

During *IP2*, anodal tDCS (DC-stimulator, Neuroconn, Ilmenau, Germany) is focally applied to the motor cortex of the lesioned hemisphere. The stimulation is applied through a 4 × 1 montage (51) where the anode is placed over the C3 (or C4) position according to the 10–20 EEG-system. The 4 cathodes are equally distanced around it and placed at the positions of CP5, CP1, FC1, FC5 (or CP6, CP2, FC2, and FC6). EEG electrodes and tDCS electrodes will be exchanged during therapy and custom-made hybrid holders have been created. The exact position of the electrodes is based on simulation results using SimNIBS 3.0 (52). In these simulations, we aimed at optimizing electrodes' location in terms of highest current density peak and broad and strong activation of primary motor and somatosensory cortices, while considering physical constraints for placing electrodes without creating bridges. The Ag/Ag-Cl electrodes (12 mm diameter) are placed, with electrolytic gel, in the EEG cap used for brain activity recording. More details on the functioning and setup of these different technologies are reported in the Supplementary Material.

#### Interventional Session

One interventional session lasts approximately 2.5 h and includes setup, calibration, therapy, and assessment of the sFM-UE, if required. The full session is performed by a trained study therapist and run from a graphical user interface (see Supplementary Material).

#### Set-Up

**Figure 2:**
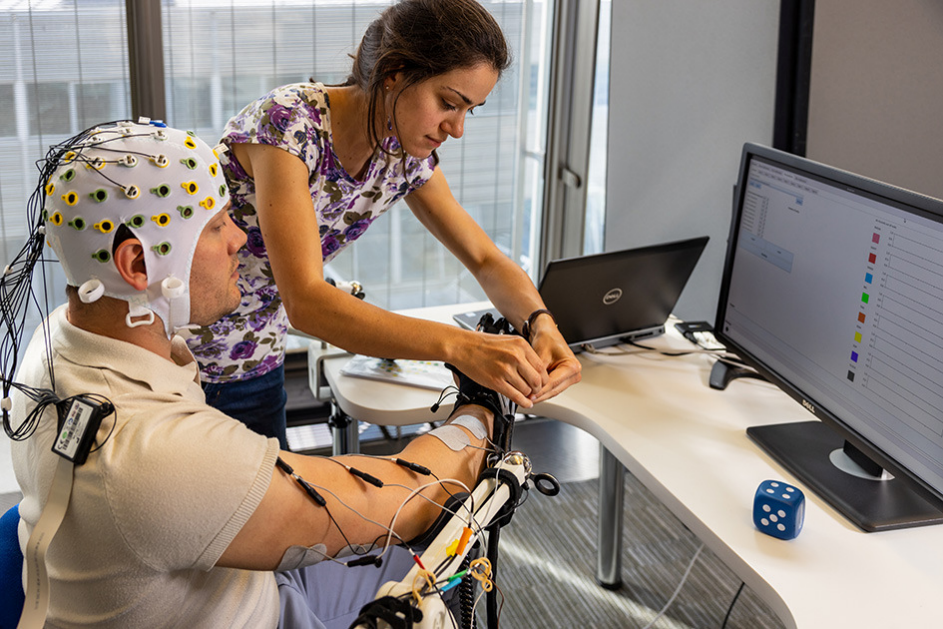
Interventional setup. The patient is sitting on a comfortable chair with the arm resting on an anti-gravity support; multiple FES electrodes are placed over the upper-limb; and the hand is placed in the robotic glove, which the therapist is adjusting. The subject is wearing an EEG cap for the BCI. On the computer screen the in-house application through which the therapy session can be run is visible.

The patient sits in a comfortable chair in front of a screen. The impaired arm is placed on an antigravity support (Armon arm, integrated with Gloreha Sinfonia system) with the elbow flexed at around 90°. Electrodes for FES are placed over the bellies of the muscles of interest and the impaired hand is inside the glove exoskeleton in anatomical rest position (Figure 2).

#### Calibration Procedure

At each new session, both the BCI and the actuators need to be calibrated. For the former, we seek a high accuracy (i.e., at least 70% offline) in classifying motor intention of the affected upper-limb. For the latter, given the synergistic work of the exoskeleton and the FES, we aim at combining them to artificially generate smooth movements that strongly resemble natural ones. This requires a fine-tuning of the actuators in terms of activation time and intensity. See Supplementary Material for further details.

#### tDCS

If in I*P2*, anodal tDCS is given at 2 mA (with 8 seconds ramp-up and ramp-down at the beginning and end respectively) for 20 min when the patient is at rest before the BCI-based rehabilitation.

#### Therapy

For each session, the study therapist chooses the exercises from a broad range of possibilities including simple movements (e.g., hand opening and closing) and functional exercises (e.g., reach and grasp a cup); the full list is available in the Supplementary Material. The patient then receives instructions on a video screen indicating the exercises that he/she has to follow. For each repetition of each exercise, as soon as the BCI decoder classifies a movement intention the actuators are initiated.

### Assessments

Longitudinal multimodal data is gathered during the different visits. The full procedure and tests can be seen in Figure 1. Baseline characteristics describing patients' demographics are performed right after the inclusion. Clinical assessments are performed at the observation visits, the full evaluation visits as well as at follow-up (i.e., 3 months after end of treatment). Neural correlates of motor impairments are performed at the full evaluation visits in T0, T1, and T2 (see Figure 1 for full procedure).

#### Observational Period

Given the absence of a control group, 10 randomized patients will also do, in addition to the standard protocol, a 3-month observational period prior to the intervention to further validate that the targeted population is in the plateau phase of recovery with a stable level of motor impairment, as suggested in the literature.

Upon recruitment, patients are randomized with a one-to-two distribution into the waiting or the starting group. The waiting group (*n* = 10) corresponds to the patients doing first the observational period and then starting the treatment phase; the starting group (*n* = 20) will directly begin with the treatment phase. The waiting group undergoes an observational period of 3 months prior to beginning the treatment, whereas the starting group begins the treatment immediately. Once the intervention starts, both groups continue the procedure in the same way and receive the same treatment described in the previous paragraphs.

The assignment of patients (waiting or starting group) is performed per center and takes age group (before 50, between 50 and 65, and over 65) and side of impairment into account (53).

#### Blinding Procedures

Complete blinding cannot be achieved in this study design (i.e., patients know in which group they have been placed – waiting vs. starting group, though the treatment phase is identical for both groups). The therapist performing the interventions is blinded to the scores given at the evaluation visits by the assessor. Within the protocol, there is at least one trained therapist per study site. The therapist assigned to one study site performs the intervention and assesses the sFM-UE for all patients belonging to the same site. An assessor, not involved in any way in the treatment (i.e., blinded to therapies' outcomes and sFM-UE) conducts the assessments at the full evaluation visits (T0, T1, and T2). The therapist of one site takes the assessor role for the other site for the clinical measures.

#### Baseline Characteristics

At baseline, descriptive information such as: age, time since stroke, affected side, medication use, and comorbidities are acquired. Furthermore, the MoCa (54), a full neurological exam, the NIHSS (41), the Cumulative Illness Rating scale (42), the Bells cancellation test (37), apraxia screening of TULIA (38), language screening test for aphasia (39), and the motricity index (40), are conducted.

#### Clinical Assessments

An assessment battery covering motor impairment at the functional, activity and participation level and several relevant other domains is conducted at each time point. The battery consists of: FM-UE (35), Action Research Arm Test (44), modified Ashworth scale (MAS) (43), Medical Research Council scale (MRC), subsections of phasic alertness and divided attention of the test of attentional performance (46), Stroke Impact Scale 3.0 (47), Beck Depression Inventory scale (48), Rivermead Assessment of Somatosensory Performance (45), and Multidimensional Fatigue Inventory (49). During the full evaluation visits (T0, T1, T2), sleep is monitored with an ActiGraph device (wGT3X-BT, Florida, USA) combined with the Pittsburgh sleep quality index (50) and a sleep diary. The FM-UE is instrumented with wearable 3D motion capture sensors (Xsens MVN, Enschede, Netherlands) and electromyography (EMG) sensors (Noraxon, Arizona, USA), and videotaped. The instrumented FM-UE can provide kinematic data and thus further evaluation of motor improvement on the same movements of the primary outcome scale; for example, information regarding smoothness and speed of movement (55, 56) will be retrieved and analyzed. Having recordings of the FM-UE will give the possibility (if necessary) to have an additional assessor evaluating the scale used for the primary outcome. For more information on the instrumented FM-UE, see the related paragraph in the Supplementary Material. Finally, an in-house self-questionnaire is used to evaluate how patients felt about the therapy and their expectancies.

#### Neural Correlates of Motor Improvement

In addition to the clinical scales, we will use multi-modal neuroimaging and electrophysiological techniques to study the underlying mechanisms of rehabilitation. We perform structural and functional (resting-state and task-related) magnetic resonance imagining (MRI). Electrophysiological parameters will be measured with EEG, transcranial magnetic stimulation (TMS), TMS-EEG and EMG. See Supplementary Material for details of the MRI-based neuroimaging and the electrophysiological protocols. Analyses of TMS/TMS-EEG evoked potential features, such as motor evoked potentials (MEPs) global and local mean field power will be determined. Regarding the neuroimaging data, factors such as the corticospinal integrity, whole brain connectivity and disconnectivity will be investigated with structural MRI; resting state fMRI allows to determine changes in resting state connectivity and with task-based fMRI, functional connectivity analyses will be performed. Electrophysiological and neuroimaging exams are performed by trained scientists and study therapists.

### Population Size

The study aims to recruit 40 patients, which by taking into account an estimated 20% dropout, leads to 30 patients completing the study with 10 starting in the waiting group (2:1 design). This number was obtained through an a-priori power analysis for sample size in G-Power (57) for a paired Wilcoxon Signed Rank test considering an alpha and beta of 0.05 and expecting an effect size of 0.64 (i.e., considering a mean FM-UE of 15, standard deviation of 4.7 (58) and expected improvement of 4 points). The sample size is also in agreement with previous neurotechnology-related studies and considering that the study is a proof of concept.

### Primary and Secondary Outcome Measures

The primary outcome measure is the change in the 66-point FM-UE. The null hypothesis will be rejected when a statistically significant improvement between pre-intervention (T0) and the end of the cumulative treatment (T2) of at least 4 points is found. A medically clinically improvement difference (MCID) lower than the commonly used 5.25 point was chosen based on three current studies addressing the MCID (59–61). The three studies involve different stroke subgroups: chronic and moderately impaired (59), subacute and moderately impaired (60), and subacute and severely-to-moderately impaired (61). As suggested by (60), being in the chronic state makes the MCID drop significantly (5 vs. 9–11 points) and further reduction (around 2 points) is observed with severity. A lower MCID is also supported by (62) that suggests a decrease in the standard error in the reliability and validity of the FM-UE with severely affected subjects as there are less uncertainties when no movement can be performed.

Secondary outcomes look at the effectiveness of the two interventional phases separately and at the general benefit of the rehabilitation compared with the natural course determined from the waiting group; the latter is expected to be negligible in the chronic state. This comparison will be done through the clinical scales evaluated during the observational period of the waiting group. To evaluate if the recovery is long-lasting, all patients will be assessed on clinical scales (including FM-UE) 3-months after T2. Moreover, to study the underlying mechanisms of motor recovery during treatment, we will base our analyses on longitudinal multi-modal assessments data: motor and cognitive scales scores will be evaluated together with neural correlates from neuroimaging techniques including MRI and a combination of electrophysiological measures, as detailed above. Secondary outcomes also include safety, feasibility, and tolerability of the experiment.

### Statistics

To evaluate if significant motor improvement of the upper-limb is achieved, the FM-UE scores of all the 30 patients will be compared between the final (T2) and the initial (T0) evaluation visits: we will use a two-tail paired Wilcoxon Signed Rank test, with the probability value set at 0.05. The null hypothesis is H0 = “FM-UE scores at T2 and FM-UE scores at T0 follow the same distribution”, H1 = “FM-UE scores at T2 do not follow the same distributions of scores at T0; the distributions are significantly different”. H0 will be rejected if a *p-*value < 0.05 is obtained. This evaluation will be considered together with the expected four-point improvement on average.

To assess if the observed changes in the FM-UE are long-lasting, we will compare with the same test (Wilcoxon Signed Rank, alpha = 0.05) the FM-UE scores at T2 with those acquired at the follow-up (3 months after T2). Another related secondary outcome regards the validation of motor function stability of chronic stroke patients (i.e., no FM-UE improvement during the observation period). We will use a Kruskal-Wallis test with alpha at 0.05 to investigate differences in the FM-UE scores at the three time points of the observation period. We do not expect to reject the null hypothesis for which the three groups are said to come from the same distribution.

Among the most important secondary outcomes is safety and feasibility; for these, descriptive statistics will be provided. One major safety outcome is incidence of epileptic seizures during the intervention period with tDCS, as well as other SAEs. Furthermore, feasibility will be determined by the drop-out rate, usually in clinical trials set to up to 20%.

Important exploratory analyses will focus on the difference between the two interventional phases; such investigation might give an insight on the role of tDCS in the rehabilitation. To test the effect of dose and intervention type on the FM-UE we will use a mixed effects linear model with random effect of subject and fixed effects of session, intervention type and the interaction of the two. If significant results will be observed, we can then build on this initial model by adding more independent variables such as lesion location and time post-stroke. Other analyses will correlate the multimodal data acquired during the three full evaluations (T0, T1, T2), such as corticospinal tract integrity and the TMS evoked potential, with the clinical scales.

Statistical analyses will be performed in a per protocol way using only the data of patients who have finished the study protocol (i.e., drop-out and excluded patients will not be included in the main analyses).

### Data and Safety Monitoring

An independent data safety and monitoring board (DSMB) is implemented and will be informed about adverse events (AE), potentially related to the use of the device, any serious adverse events (SAE), device deficiencies, and the progress of the trial. (S)AEs follow the definition of ISO 14155:2020 3.2 and 3.45. All SAE and device deficiencies will be reported to the Sponsor within 24 h. The Sponsor is obliged to report to the former to the Competent Ethics Committee (Commission cantonale d'éthique de la Recherche sur l'être humain in Vaud) and the second one to Competent Authority (Swissmedics) within 7 calendar days. The trial will be stopped if there is a medically relevant increase in major, unexpected AE with the intervention compared with the waiting group. Moreover, the study has implemented a monitoring plan involving 10 site visits, including the site-initiation and close-out visits. The monitoring will cover the conduct of the study, the completeness of files and documents as well as data security, (S)AE and device deficiencies.

## Discussion

The AVANCER protocol proposes two important novelties to enhance neurorehabilitation and bring forward the current therapeutic options in severely impaired stroke patients: a combination of neurotechnologies and a highly personalized treatment intervention. We believe that the present interventional strategy, combining several neurotechnologies in a personalized fashion, will have a large potential to be translated into clinical settings, if successful. We hypothesize that the personalized, sequential multi-technology-based interventions will further support behavioral restitution of impaired functions in this group of patients with limited treatment options in the chronic stage. We expect that this treatment will mechanistically not only act on brain plasticity and functional reorganization (BCI and tDCS), but will also show beneficial effects on the musculoskeletal system in a synergistical way. Specifically, with the support of FES and exoskeleton, a multitude of repetitive upper extremity movements can be performed by the patient allowing full upper limb movements (not possible in these patients without the present interventional strategy). Such movements are performed thanks to an exoskeleton that passively extends and flexes the fingers and supra-motor threshold FES on seven muscles. The continuous movements might help decreasing possible spasticity, atrophy and hopefully reverse some of the changes that resulted from months of no movements performed.

Regarding brain plasticity, previous BCI studies have reported cortical reorganization alongside the clinical motor improvement (19, 20, 63). These results are thought to be partly due to the brain engagement needed for the BCI to work (64) and we hypothesize that the underlying plastic changes can further be enhanced with tDCS, which was reported to have a positive effect on the strength of event related desynchronizations (65–67) and motor outcome (68, 69). We assume that behavioral restitution of function, induced by the combined neurotechnology approach will go alongside with cortical reorganization processes, which we plan to capture by a multimodal evaluation by means of (f)MRI, DTI and TMS-EEG related outcome measures. These combined measures will allow to show if the present approach can induce changes on multiple levels of the phenomenological model (70) underlying recovery after stroke.

The study outcome is evaluated with the 66-point FM-UE, currently seen as the gold standard measure for motor impairment and function of the upper extremity. Following the suggestions of the SRRR (28), we also instrument this scale with EMG and IMUs to gather further data such as kinematic information, able to better characterize movement quality and functional changes.

The expected primary outcome is a four-point improvement in the FM-UE on average across patients and this is set to be the MCID in this study. Although this is lower than previously suggested (59), it is of note that this suggestion was based on less impaired stroke patients in the acute phase post-stroke, we believe it is an appropriate MCID for the present population of severely impaired patients (60, 61).

The FM-UE, together with the other longitudinal assessed scales and neural correlates will provide important data for understanding the effects of the intervention at a group level and for identifying patients who will potentially benefit from this intervention.

## Summary and Conclusion

The experimental and technological set-up of the present clinical trial, where an exoskeleton, multi-electrode FES, BCI and non-invasive brain stimulation are used together in a personalized fashion in a clinical trial aimed at improving upper limb impairment in severely affected stroke patients with so far very limited treatment options. We propose a personalized, patient-tailored interventional strategy, where the targeted population is provided with advanced neurotechnologies to achieve maximized interventional effects, without restraining with one type of intervention, but rather have an add-on, synergistic continuous therapy following the individual needs and improvement. We aim to achieve motor improvement due to the central engagement given by BCI and later enhanced by tDCS and the peripheral activation of natural afferent and efferent pathways by the concomitant action of actuators and further enriched through the performance of a variety of functional exercises. The trial will allow the collection of longitudinal multi-modal neurophysiological data constituting a precious dataset for studying motor recovery in the target population.

## Ethics Statement

This study has been approved by the Ethics Committee of the Canton of Vaud (No. 2019-00094) and Swissmedic (No. 10000577). The patients/participants provided their written informed consent to participate in this study.

## Author Contributions

MC, FH, and NB designed the initial study. ClB, AC, and AE developed the setup. SZ, TH, JH, and MO contributed in the therapeutic aspect of the protocol. ChB drafted the first version of the manuscript. GE ran the tDCS simulations. MW, SZ, and FH helped in choosing the tDCS stimulation. All authors contributed to the final protocol, read, revised the paper, and approved the submitted version.

## Funding

AVANCER is a sponsor-initiated trial, which received funding from the Wyss Center for Bio and Neuroengineering (WCP-030). Sponsor is the Wyss Center for Bio and Neuroengineering. It is partially supported by #2017-205 “Personalized Health and Related Technologies (PHRT-205)” of the ETH Domain and the Defitech Foundation (Morges, Switzerland). Open access funding provided by École Polytechnique Fédérale de Lausanne.

## Conflict of Interest

MC and AE is employed by the Wyss Center acting as the sponsor of this study. MC is employed by confinis AG. The remaining authors declare that the research was conducted in the absence of any commercial or financial relationships that could be construed as a potential conflict of interest.

## Publisher's Note

All claims expressed in this article are solely those of the authors and do not necessarily represent those of their affiliated organizations, or those of the publisher, the editors and the reviewers. Any product that may be evaluated in this article, or claim that may be made by its manufacturer, is not guaranteed or endorsed by the publisher.

## Footnotes

^1^Cerebrovascular accident and contribution of individualized neurotechnologies for severely impaired patient: a prospective clinical trial aiming at restoring the upper-limb mobility.
