# Supplementary material for: A Novel Patient-Tailored, Cumulative Neurotechnology-Based Therapy for Upper-Limb Rehabilitation in Severely Impaired Chronic Stroke Patients: The AVANCER Study Protocol
Source: Front Neurol. 2022 Jul 7;13:919511. doi: 10.3389/fneur.2022.919511 (PMC9301337; doi:10.3389/fneur.2022.919511)
Supplement: Supplementary file 1 [file Data_Sheet_1.docx]

A novel patient-tailored, cumulative neurotechnology-based therapy for upper-limb rehabilitation in severely impaired chronic stroke patients: the *AVANCER* study protocol

# Graphical user interface

The software used to govern the different devices, with the exception of tDCS, is developed in Python 3.7. To ease the procedure, we created a graphical user interface (GUI) to attend to the different steps of a session: patient selection, calibration of exoskeleton and functional electrical stimulation (FES), fine calibration of exercises combining the two actuators, tuning and training of the brain-computer interface (BCI) decoder and selection of exercises for the current session. Each step is done on a specific window of the GUI. Safety measures to avoid co-activation of different elements of the GUI and warnings related to the connected devices are implemented.

# Calibration

The calibration of each actuator is done through specific windows of the GUI, one for each device (Fig. 1, Supplementary Material).

## Exoskeleton

The Gloreha Sinfonia (Idrogenet, Brescia, Italy) robotic glove is able to flex and extend each finger independently by elongating or shortening five mechanical wires placed into the guides of the glove worn by the subject [1]. In addition, it has embedded stretch sensors for each finger. Therefore, two types of calibrations are possible: sensor calibration and wire calibration. The former is used to evaluate the active and passive range of motion (ROM) where fingers are moved by the patient or by the therapist, respectively. The latter is needed to tune the flexion of the fingers for each exercise. The combination of the two calibrations can be used to perform assisted-as-needed movements. With this approach, the glove does not passively perform the movement immediately, but waits for an active movement of the fingers for several seconds. If a movement of at least one fifth of the active ROM is detected, the exoskeleton will close or open the hand by the same fraction of ROM. If no movement is detected, the glove will passively perform the full movement.

The maximum extension of each finger is fixed manually, and all the flexions are calibrated through the software as a percentage of it: the maximum extension is always seen as 100% by the software. The rest anatomical position is also tuned through software as for the exercise flexions (Fig. 1a Supplementary Material).

## Functional electrical stimulation (FES)

The maximum intensity (mA) to have an active contraction of the muscle of interest is adjusted in the FES-dedicated window of the GUI. Here, each muscle and associated channel can be configured independently. When the appropriate intensity is found, it is saved to all the exercises (Fig. 1b Supplementary Material).

## FES and exoskeleton

Fine-tuning of all the exercises that combine the two actuators is achieved through the time-planner window in which the timing of each actuator can be configured. Here, each channel of the FES is considered independently, whereas fingers are considered as a whole, i.e., the glove. Each exercise has a predefined default, which defines the duration of the exercise and the subdivision of the exercise into sequential tasks. Each task has the following properties for each channel: 1) ON/OFF; 2) initial position (or intensity); 3) final position (or intensity); 4) time of beginning of transition (decimal of second precision; i.e. time-point in subtask when the intensity or ROM start changing for the FES or glove respectively); 5) time of end of transition (decimal of second precision; i.e. time-point in subtask when the intensity or ROM stop changing for the FES or glove respectively). See Fig. 1c SM. Exercises start and finish with the subject in rest position, and with the FES stimulation turned off. An example of exercise with tasks division is shown in Fig. 2 Supplementary Material.

## Brain-computer interface (BCI)

As for the actuators, the BCI has its own calibration window in the GUI (Fig. 1d SM). The calibration of the decoder (i.e., the classifier) requires a labelled training set, which is acquired at each therapy session by asking the patient to perform three actions for a minimum of 20 repetitions: move the affected hand, move the non-affected hand, and relax. These instructions are given from a screen placed in front of the patient and are synchronized with triggers sent to the EEG amplifier. If moving of the affected hand is not possible, the patient is instructed to try to move, without making compensatory movements, even if this attempt does not lead to an actual movement.

Once this labelled dataset is gathered, a machine learning algorithm based on linear discriminant analysis is trained to find the features that best discriminate the “move the affected hand” class from the “relax” one. The class “move the non-affected hand” is acquired for post-hoc analyses of lateralization, but it is not used for the online model. The features to decode the different classes are based on the power spectral density (PSD) of the ipsilesional channels (i.e., nine channels over the motor cortex of the ipsilesional hemisphere) at each frequency band (2Hz wide) ranging from 2 to 40 Hz. Features, as well as hyper-parameters, are tuned using cross-validation.

The offline results (i.e., performance of the classifier based on the training set alone) aid in choosing whether to save or discard the current model. A model is considered good if at least 70% accuracy for both classes is reached. If the model has a good offline performance, it is then tested online in the validation phase. Similarly to the model training, the patient will be asked to move the affected hand and rest for at least 10 repetitions. On top, neurofeedback can be added at patient discretion. This is used to give online feedback to the patient and is in the form of a bar, whose height changes according to the probability that the current signal (i.e. movement intention vs. rest) is classified correctly by the automatic decoded.

# List of exercises

The combination of multiple-channel FES and exoskeleton allowed us to create a broad list of movements. The full list is reported hereunder; some are simple movements involving only few joints (e.g., shoulder flexion), while others can be considered as full exercises (e.g., reach and grasp an object).

- Shoulder flexion (only FES),
- Shoulder flexion with supination,
- Shoulder abduction (only FES),
- Shoulder abduction with elbow extension
- Hand to mouth (only FES),
- Hand to mouth with grip,
- Hand to mouth with grasp,
- Hand to mouth with pinch,
- Wrist dorsiflexion,
- Hand opening and grasp,
- Hand opening and pinch,
- Hand opening and tripod pinch,
- Hand opening and cylindrical grip,
- Finger counting,
- Finger tapping,
- Reach and grasp a cup,
- Reach and grasp a pen,
- Reach and grasp a ball,
- Clean the table.

1. **Stimulation for electrodes placement for high definition focal tDCS**

The gold standard montage for tDCS over the motor cortex is considered to be the large sponge-based setup, with the active electrode placed at the motor cortex and the return electrode on the frontal contralateral cortex with a surface area of 25cm^2^, and a current intensity of 1mA [2]. In the last decade, novel montages have appeared. Among others, the high definition montage has already been used in several clinical studies [3–9]. In this montage the active electrode, with a small surface area is placed on the target region with multiple return electrodes on a fixed distance surrounding it [7]; because the electrodes used have a rather small diameter (10-20mm), they provide focal stimulation.

The exact positioning of the high definition 4x1 montage electrodes is however not fixed and we used simulations to find the best placement of all of them, using as benchmark the gold standard. Simulations were done using SimNIBS 3.0 [10] which computes the electric field and current density induced by transcranial stimulation using Finite-Element Methods. Specifically, we aimed at positioning Ag/AgCl lentil electrodes, as developed for the DC-stimulator (Neuroconn, Ilmenau, Germany). As starting position we placed the anode (i.e., central electrode) near C3, and the four cathodes around it at the same distance, rotated at 45 degrees. From there, we manually translated the anode around the motor cortex, as well as rotated the cathodes around it to evaluate if major differences were obtained in the induced electric field. Different distances between the anode and the cathodes were also tried. The goal of this optimization was to achieve focal motor and sensory cortices stimulation, and a high current density peak in those regions. The final choice was then restricted by the physical impossibility of cutting the EEG cap too close to EEG electrodes needed for the BCI.

Fig. 3a Supplementary Material, shows the results of the gold-standard montage. The active electrode is positioned over the left motor cortex (C3 in the international 10-20 EEG system) with an orientation of approximately 45° to the midline. The return electrode is placed over the right frontal cortex (F4 international 10-20 system). Current intensity was 1 mA. Fig. 3b Supplementary Material, shows the simulation results with the chosen 4x1 montage, where the anode is placed over C3 and the 4 cathodes are around it at CP5, CP1, FC1, FC5. Current intensity is 2 mA. We can observe that the current density peak is in both cases far below the presumed threshold for tissue damage [11].

# TMS-EEG protocol

At T0, T1 and T2 the patient will also attend a transcranial magnetic stimulation (TMS)-EEG session. During the experiment, the subject will be seated in a comfortable chair. We will record EEG activity through 64 Ag/AgCl TMS-compatible electrodes in a 10-20 system (ActiCap 64 channels, Brainvision, Gilching, Germany). Sampling rate will be put at 2.5 kHz, with a high cut-off of 1 kHz. The reference will be placed at AFz and the ground at Fpz. Electromyography (EMG) activity will be acquired using a pair of disposable Ag-AgCl electrodes. The signal will be amplified and sampled at 3 kHz using a Noraxon DTS Receiver (Scottasdale, Arizona, United States) with the band-pass filter from 10 Hz to 1000 Hz (analog Sallen-Key for high-pass filter and digital FIR filter with order 128 for the low-pass; the gain will be set at 500), and will be digitized at 5 kHz using Signal software (Cambridge Electronic Design Limited, Cambridge, UK) for further processing on a laptop. Seven muscles on the affected upper limb will be recorded throughout the experiment: the first dorsal intraossei (FDI), abductor digitor minimi, abductor pollicis brevi, flexor carpi ulnaris, flexor carpi radialis, extensor carpi radialis longus and brevis, extensor carpi ulnaris. The FDI of the non-affected hand will also be recorded. Transcranial magnetic stimulation will be delivered with a MagPro X100 stimulator connected to an MC-B70 coil (Magventure, Farum, Denmark). Moreover, stimulation will be applied using Neuronavigation (Localite GmbH, Bonn, Germany) along all the experiment. This will use the T1 image of the patient previously acquired (see (f)MRI paragraph on SM for specifics).

A minimum of four and a maximum of six stimulation blocks will be delivered at every session. Each block will be composed of thirty single-pulse and thirty short-interval intracortical inhibition (SICI) pulses placed in a pseudo-randomized manner; two pseudo-randomized sequences will be used. The stimulation location will be the FDI hotspot of the affected hand. This will be searched on the affected hemisphere as the location that elicits the highest motor-evoked potential (MEP). If no MEP can be elicited, the hotspot will be chosen from the anatomy as the hand-knob of the affected hemisphere. Stimulation intensities will be found from the non-affected hemisphere. Single pulses will be delivered at the test intensity, which is the intensity consistently giving an MEP with peak-to-peak amplitude of at least 0.5mV. SICI will also be delivered at the same intensity, with the conditioning stimulus at 80% of the resting motor threshold (RMT). The latter is defined as the intensity that elicits an MEP with peak-to-peak amplitude of at least 0.05mV five times out of 10. Pulses will be applied every 4 seconds with a 25% jitter. During the stimulation blocks, patients will be wearing noise-cancelling headphones providing white noise to cover the “click” provided by the stimulator.

Before and after the stimulation blocks we will acquire three minutes of resting state with eyes open (fixating a cross) and eyes closed.

# (f)-MRI protocol

The imaging session will be performed in a 3T MAGNETOM PRISMA scanner (Siemens, Erlangen, Germany). Structural T1-weighted images will be acquired by 3D MPRAGE sequence with the following parameters: TR = 2.3s; TE = 2.96 ms; flip angle = 9°; slices = 192; voxel size = 1 × 1 × 1 mm, FOV = 256mm. T2 images will be also collected with the following parameters: TR = 3s; TE = 409 ms; slices = 208; voxel size = 0.8 × 0.8 × 0.8 mm, FOV = 256mm. Echo-Planar Imaging (EPI) sequences will be used to obtain resting-state and task-based functional images. Resting state sequences will be acquired with the following parameters: TR = 1.25s; TE = 32ms; flip angle = 58°; slices = 75; voxel size = 2 × 2 × 2 mm; FOV = 224mm. Task-based images will be obtained by applying the following parameters: TR = 1s; TE = 32ms; flip angle = 50°; slices = 66; voxel size = 2 × 2 × 2 mm; FOV = 224mm. Diffusion-weighted images (DWI) will be obtained by collecting seven T2-weighted images without diffusion weighting (b0;b=0s/mm2), including one in opposite phase encoded direction, and 101 images with noncollinear diffusion gradient directions distributed equally over the half-sphere covering 5 diffusion gradient strengths (b-values=[300.0,700.0,1000.0,2000.0,3000.0]s/mm2; shell-samples=[3,7,16,29,46]). These images will be acquired using pulsed gradient spin echo technique, with the following parameters: TR = 5000ms; TE = 77ms; slices = 84; voxel size = 1.6 × 1.6 × 1.6mm; FOV = 234mm. No medication or contrast agents will be given.

The assessment protocol will consist in around 8 minutes of resting state data, around 9 minutes of visual control task, around 9 minutes of motor task and additional structural sequences. During the motor task participants are required to attempt to perform or perform hand force generation involving the paretic and non-paretic hand. For this, the participant is first provided with the gripper(s) to be used in the task and instructed to look at an initially black screen and follow the instructions. The first set of instructions requires the participant to let go of the trigger activating the gripper while holding the device in a comfortable position. A relaxation value is obtained at this time, to serve as a lower bound during the task. The participant is then instructed by a message on the screen to exert and hold as much force possible on the gripper. An average of the applied maximum force is calculated over three repetitions. After the rest and maximum force values have been obtained, the participant may start with the task. During the task, two concentric rings are presented, with a fixation cross at their center. The rings, normally gray, are given a white shade to indicate the target ring. An additional ring (dark gray, here referred to as the output ring), changes its size proportionally to the force applied by the participant. The goal of the participant is, then, to match the size of the output ring to that of the white, target ring. The outer ring corresponds to no force applied, whereas the inner ring is reached when applying a preset percentage of the maximum voluntary contraction (i.e. 20%). The target rings switch continuously so that the participant needs to apply force and release rhythmically over time. If participant have not enough force to perform the task, a standard visual feedback is shown with the theoretical movement of the output ring. Rest periods (during which only the fixation cross is shown) are allowed between blocks. The visual control condition consists of the same display of switching target rings, with the difference of the participant being instructed not to apply force at all (the output ring is not shown), but only to look at the ring which becomes white.

# Instrumented Fugl-Meyer Assessment protocol

Recently the Stroke Recovery and Rehabilitation Roundtable [12] suggested the potential of kinematic measures to evaluate motor improvement. Following the increased interest in the topic, we decided to instrument the assessment of our primary outcome: the Fugl-Meyer Assessment of the upper limb (FM-UE) [13]. The assessment will see the patient wear wireless EMG sensors as well as inertial motor units (IMUs) (Xsens MVN, Enschede, Netherlands). The former will be placed to cover the following muscles of both upper limbs: trapezius, deltoid, biceps long head, teres major, triceps, pectoralis, pronator teres, extensor carpi radialis, flexor ulnaris. The signal will be amplified and sampled at 3 kHz using a Noraxon DTS Receiver (Scottasdale, Arizona, United States) with the band-pass filter at 10 Hz to 1000 Hz (analog Sallen-Key for high-pass filter and digital FIR filter with order 128 for the low-pass; the gain will be set at 500). The 3D motion capture sensors based on IMUs will be place on the forehead, both hands, both wrist and upper arms (placed right below the deltoids), on the sternum and on the sacrum, at the level of S1; on the shoulders at 1cm lateral from the scapula border and on top of the spina scapula. Following the instructions from the manufacturer [14], a calibration step will be performed before starting the real assessment. The Noraxon receiver will receive a trigger as soon as the Xsens software begins and ends to record the data. The assessment will also be videotaped. For each FMA item, the patients will be asked to perform it five times.

# References

[1] Borboni A, Villafañe JH, Mullè C, Valdes K, Faglia R, Taveggia G, et al. Robot-Assisted Rehabilitation of Hand Paralysis After Stroke Reduces Wrist Edema and Pain: A Prospective Clinical Trial. Journal of Manipulative & Physiological Therapeutics 2017;40:21–30. https://doi.org/10.1016/j.jmpt.2016.10.003.

[2] Zimerman M, Heise KF, Hoppe J, Cohen LG, Gerloff C, Hummel FC. Modulation of Training by Single-Session Transcranial Direct Current Stimulation to the Intact Motor Cortex Enhances Motor Skill Acquisition of the Paretic Hand. Stroke 2012;43:2185–91. https://doi.org/10.1161/STROKEAHA.111.645382.

[3] Zandvliet SB, Meskers CGM, Kwakkel G, van Wegen EEH. Short-Term Effects of Cerebellar tDCS on Standing Balance Performance in Patients with Chronic Stroke and Healthy Age-Matched Elderly. Cerebellum 2018;17:575–89. https://doi.org/10.1007/s12311-018-0939-0.

[4] Edwards D, Cortes M, Datta A, Minhas P, Wassermann EM, Bikson M. Physiological and modeling evidence for focal transcranial electrical brain stimulation in humans: A basis for high-definition tDCS. Neuroimage 2013;74:266–75. https://doi.org/10.1016/j.neuroimage.2013.01.042.

[5] Hill AT, Rogasch NC, Fitzgerald PB, Hoy KE. Effects of single versus dual-site High-Definition transcranial direct current stimulation (HD-tDCS) on cortical reactivity and working memory performance in healthy subjects. Brain Stimul 2018;11:1033–43. https://doi.org/10.1016/j.brs.2018.06.005.

[6] Kindred JH, Kautz SA, Wonsetler EC, Bowden MG. Single Sessions of High-Definition Transcranial Direct Current Stimulation Do Not Alter Lower Extremity Biomechanical or Corticomotor Response Variables Post-stroke. Front Neurosci 2019;13:286. https://doi.org/10.3389/fnins.2019.00286.

[7] Kuo H-I, Bikson M, Datta A, Minhas P, Paulus W, Kuo M-F, et al. Comparing Cortical Plasticity Induced by Conventional and High-Definition 4 × 1 Ring tDCS: A Neurophysiological Study. Brain Stimulation 2013;6:644–8. https://doi.org/10.1016/j.brs.2012.09.010.

[8] Minhas P, Bansal V, Patel J, Ho JS, Diaz J, Datta A, et al. Electrodes for high-definition transcutaneous DC stimulation for applications in drug delivery and electrotherapy, including tDCS. J Neurosci Methods 2010;190:188–97. https://doi.org/10.1016/j.jneumeth.2010.05.007.

[9] Boratyn D, Ruffini G, Cortes M, Rykman A, Medeiros A, Pascual-Leone A, et al. P 172. Focal tDCS in Chronic Stroke patients: A pilot study of physiological effects using TMS and concurrent EEG. Clinical Neurophysiology 2013;124:e146–7. https://doi.org/10.1016/j.clinph.2013.04.249.

[10] Thielscher A, Antunes A, Saturnino GB. Field modeling for transcranial magnetic stimulation: A useful tool to understand the physiological effects of TMS? Annu Int Conf IEEE Eng Med Biol Soc 2015;2015:222–5. https://doi.org/10.1109/EMBC.2015.7318340.

[11] Antal A, Alekseichuk I, Bikson M, Brockmöller J, Brunoni AR, Chen R, et al. Low intensity transcranial electric stimulation: Safety, ethical, legal regulatory and application guidelines. Clin Neurophysiol 2017;128:1774–809. https://doi.org/10.1016/j.clinph.2017.06.001.

[12] Bernhardt J, Hayward KS, Kwakkel G, Ward NS, Wolf SL, Borschmann K, et al. Agreed definitions and a shared vision for new standards in stroke recovery research: The Stroke Recovery and Rehabilitation Roundtable taskforce. International Journal of Stroke 2017;12:444–50. https://doi.org/10.1177/1747493017711816.

[13] Fugl-Meyer A, Jääskö L, Leyman I, Olsson S, Steglind S. The post-stroke hemiplegic patient. 1. a method for evaluation of physical performance. Scandinavian Journal of Rehabilitation Medicine 1975;7:13–31.

[14] MVN User Manual. User Guide MVN, MVN BIOMECH MVN Link, MVN Awinda. Rev. March, 2017, Xsens Technologies B.V

# Figures Captions

Fig. 1 Supplementary Material - AVANCER GUI windows screenshots for calibration of neurotechnologies: a) exoskeleton calibration; b) FES calibration; c) single task calibration; d) BCI calibration

Fig. 2 Supplementary Material - Example of task division for exercise “Hand to mouth”. a) GUI display showing the sequential (de-)activation of different channels. b) Video-clips of actual tasks performed with multi-channel FES. Task names are written at the top, activated channels at the bottom and default task duration in the lowest line. Rhom=rhomboids; dant: deltoids anterior; bic= biceps; sup=supinator muscles (i.e., brachioradialis); edm=extensor digitorum medialis.

Fig. 3 Supplementary Material - Brain stimulation simulation results. On the right side, current density induction result with color scale; on the left electrodes positioning over the brain and the 10-20 system. a) Gold-standard electrode positioning; the maximum peak intensity reached is 0.126A/m^2^ for an input current of 1mA; b) Chosen focal high-density montage; the maximum peak intensity reach is 0.126A/m^2^ for an input current of 2mA.
