# Supplementary material for: A Novel Patient-Tailored, Cumulative Neurotechnology-Based Therapy for Upper-Limb Rehabilitation in Severely Impaired Chronic Stroke Patients: The AVANCER Study Protocol
Source: Front Neurol. 2022 Jul 7;13:919511. doi: 10.3389/fneur.2022.919511 (PMC9301337; doi:10.3389/fneur.2022.919511)
Supplement: Supplementary file 2 [file Data_Sheet_2.DOCX]

# Information form for the *AV*AN*C*ER study

**A**ccident **V**asculaire cérébral et **A**pport des **N**eurotechnologies individualisées chez la personne avec une hémiparésie **C**hronique : une **E**tude clinique prospective visant à **R**estaurer la mobilité du membre supérieur. This study is organized by the Wyss Center for Bio- and Neuroengineering.

Dear Sir or Madam,

We invite you to participate in our research project.

Your participation is completely free. All data managed as part of this project is subject to strict data protection rules.

The research project is conducted by Wyss Center for Bio and Neuroengineering (promoter). We will communicate the results to you if you wish.

During an interview, we will explain to you the main points and answer your questions. To give you an overview of the project, here are the key points to remember.

You will find more detailed information below.

Why are we conducting this research project ?

- In the presence of chronic hemiparesis caused by a stroke, rehabilitation is performed in order to improve the restoration of mobility of the affected limb.
- Our research project aims to study whether the efficiency presented by our system comprising a brain-machine interface (BCI), robotic support, and muscle and brain stimulation is equivalent or superior to conventional rehabilitation.

What should I do if I agree to participate ? - What happens to me if I participate?

- Type of participation: If you agree to participate in our project, you will undergo different types of visits: assessment and clinical observation visits; full evaluation visits; and intervention visits targeted at rehabilitation.
- Procedure for the participants: participants are randomly divided into 2 groups, Group A and Group B. Whatever the group, the treatments are exactly the same. Group B participants attend more evaluation and observation visits than Group A participants, in order to assess the natural progress of their upper limb mobility.
- Duration and number of visits: The length of your participation will depend on your response to the treatment, and on the assigned group. There will be at least 22 intervention visits, plus calibration visits until you are able to use the BCI. In addition, there are up to 6 full evaluation visits and one follow-up visit.

What are the benefits and the risks related to participation in the project?

**Benefits for the participants**

- There is no guarantee that your participation in this study will bring you any benefit.
- By taking part in the project, you contribute to helping future patients who suffer from chronic stroke.

**Risks and constraints**

Your participation in the study results in risk-taking related to certain examinations, to the use of the investigational device, and to the frequency of visits. These risks involve:

- Fatigue linked to the frequency and duration of visits
- The main risks associated with the use of the investigational device:
  - A potential excessive force of the robot on the user's hand;
  - The excessive use of electrical stimulation and robotic gloves, which could cause skin irritation, muscle pain, fatigue, dermatitis, tendonitis, or muscle contractures;
  - During brain stimulation, a slight tingling or itching sensation may be felt at the site of the electrode position;
- The MRI is a noisy environment which may cause discomfort linked to noise and a slight feeling of claustrophobia;
- Transcranial magnetic stimulation (TMS) may cause slight transient headaches or moderate fatigue.
- The use of electrodes at each visit to record brain or muscle activity could cause slightly unpleasant sensations in the skin and it requires washing your hair at the end of the visit to remove the gel. Applying the electrodes to the arm may require the arm to be shaved.
- Depilation and the use of electrodes can irritate the skin of people with very sensitive skin.
- No data are yet available on the effects of the investigational device on the fetus and some of the planned tests are also contraindicated for pregnant women. Pregnancy is an exclusion criterion.

By signing at the end of the document, you certify that you have understood all the content and that you freely consent to take part in the project.

## Detailed information

## Aims of the study

In this information sheet, the research project is also referred to simply as *study*. If you agree to take part, you are a study *participant*.

We are proposing you to participate in a clinical study on the optimization and individualization of upper limb rehabilitation in patients who have suffered a stroke. We are conducting this study to verify the safety and the effectiveness of a treatment based on a set of innovative technologies : brain-computer interface (BCI), robotic support, muscle and brain stimulation. The purpose of this study is to verify the safety of the rehabilitative treatment, as well as to optimize and individualize rehabilitation of the paralyzed upper limb in patients who have suffered a stroke and for whom upper limb mobility is very limited. Clinical and psychomotor examinations carried out as part of the study will provide a better understanding on the mechanisms of success or unsuccess of the rehabilitation, with the aim of improving future therapies.

The investigational medical device is composed by the following elements:

- A brain-machine interface, including a cap with electrodes (EEG, Figure 1), and software. The software detects the patient's motor intention in real time and controls other parts of the device.
- Electrodes on the arm and wrist, for electrical muscle stimulation (FES, Figure 1).
- A robot (Gloreha Sinfonia, Figure 1) to guide the movements of the fingers in synchronization with the participant's intention.
- Computers to control the entire system.
- Electrodes on the skull for a short time, for electrical brain stimulation.


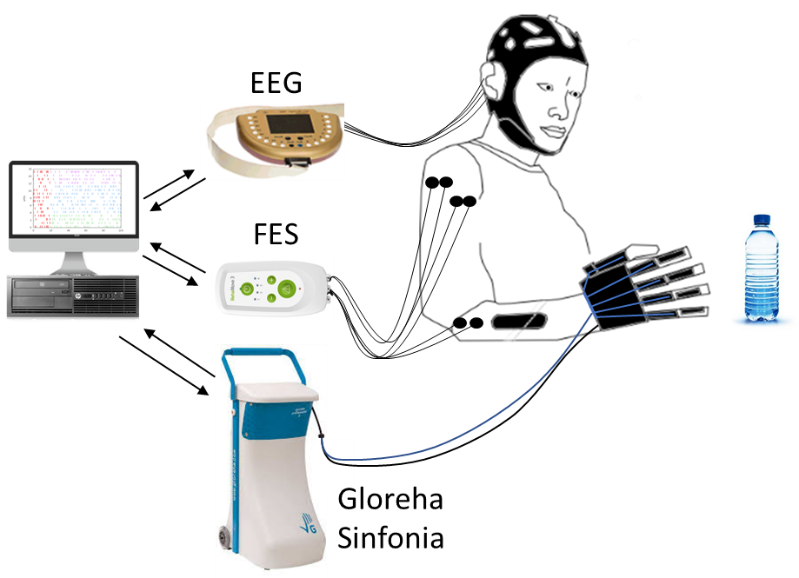

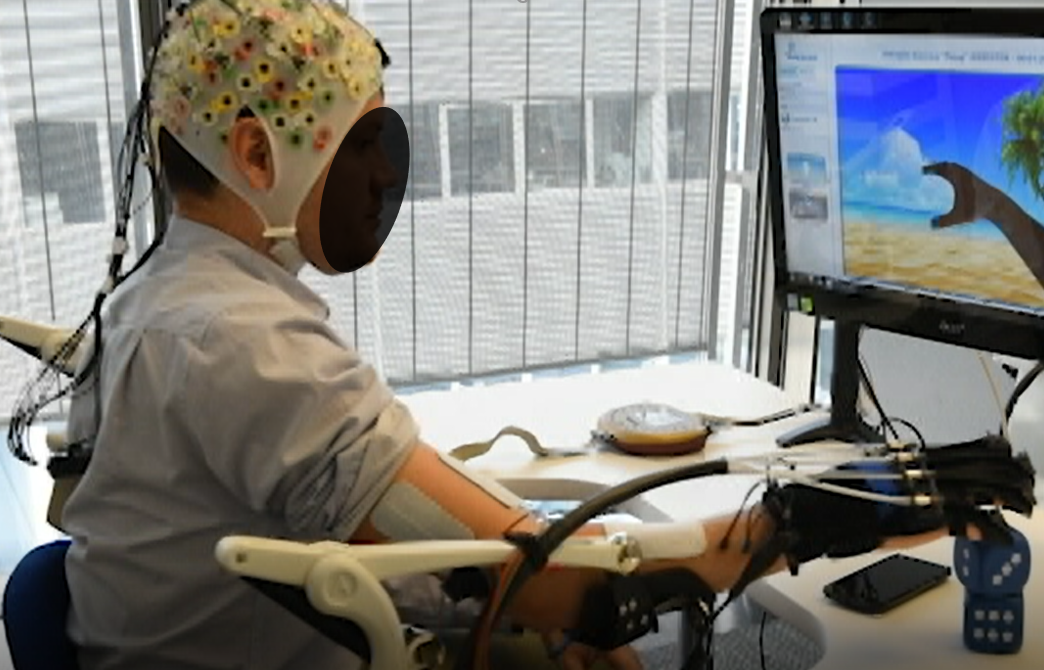


Figure 1. Schematic representation of the experimental set-up and its installation at the Campus Biotech.

## Selection of participants.

We are proposing you to participate in this study because you are 18 years old or older, you had a single stroke more than 6 months ago, and you cannot extend your fingers. You do not have severe spasticity (i.e., having a score greater than 3 on the Modified Ashword scale), and you are not participating in other clinical trials.

If you are concerned by one of the following criteria, please inform the investigator, because you will not be able to participate in the study: you have an active implant (pacemaker, etc...), you have dependency with medications or alcohol, you have psychotic symptoms, you are treated with neuroleptic or lithium medications, you are unable to understand instructions, you suffer severe neglect, you have severe upper limb rigidity that prevents the use of the robotic component of the device, you have physical characteristics that prevent the recording of brain signals or muscle stimulation (voluminous hair and large amount of subcutaneous fat in the arms), you are allergic to latex, you have dermatitis, you are involved in another clinical trial or you have to travel more than 70 km to reach one of the study sites, you are pregnant.

## General information about the study

This study will be carried out in Switzerland at the Clinique Romande de Réadaptation (Suva Care) in Sion and at the Campus Biotech in Geneva. Up to forty subjects will be included. The study has two research groups: Group A and Group B. Each participant will be assigned to one of the two groups for the entire duration of the study. Regardless of the group, the treatments are exactly the same. The participants of Group B attend more assessment and observation visits at the beginning of the study than the participants in Group A, in order to assess the natural progress of their upper limb mobility. The assignment to the groups will be done by randomization when you register, with a probability of 66.5% for group A and 33.5% for group B.

If your upper limb mobility progresses with the treatment, the intervention will be individualized and may last longer, extending your participation in the study. The length of your participation will depend on your response to the treatment, and on the group of assignment. As long as you will continue to show progress in the mobility of your affected upper limb, the treatment will continue (up to a maximum of 6 months of treatment). The total duration of the study is three years.

The treatment proposed in this study does not replace any other. You may continue any other rehabilitation or medical treatment prescribed to you in parallel, if it does not include devices similar to those included in the investigational device. We ask you to inform the investigators of the study about any other treatment you are doing. If you participate in this clinical trial, you must not participate in any other clinical trial.

We are conducting this study in compliance with Swiss legislation. In addition, we follow all internationally recognised guidelines. The cantonal ethics commission and the competent authority (Swissmedic) authorised and monitor the study. You will also find a description of the study on the website of the Federal Office of Public Health: [www.kofam.ch](http://www.kofam.ch).

## Procedure for participants

- Following inclusion, previous MRIs will be checked, if existing, otherwise a short structural MRI session will be performed in order to confirm the inclusion criteria.
- The study includes three types of visits to the research centers: **assessment and clinical observation visits** (in green in figure 2), lasting maximum 2 hours; **full evaluation visits** (in orange in Figure 2), lasting cumulatively maximum 9 hours. Given the length of the full evaluation, it generally takes place over 3 separate days; and **intervention visits** (in gray in Figure 2) for rehabilitation, lasting around 2 and half hours.


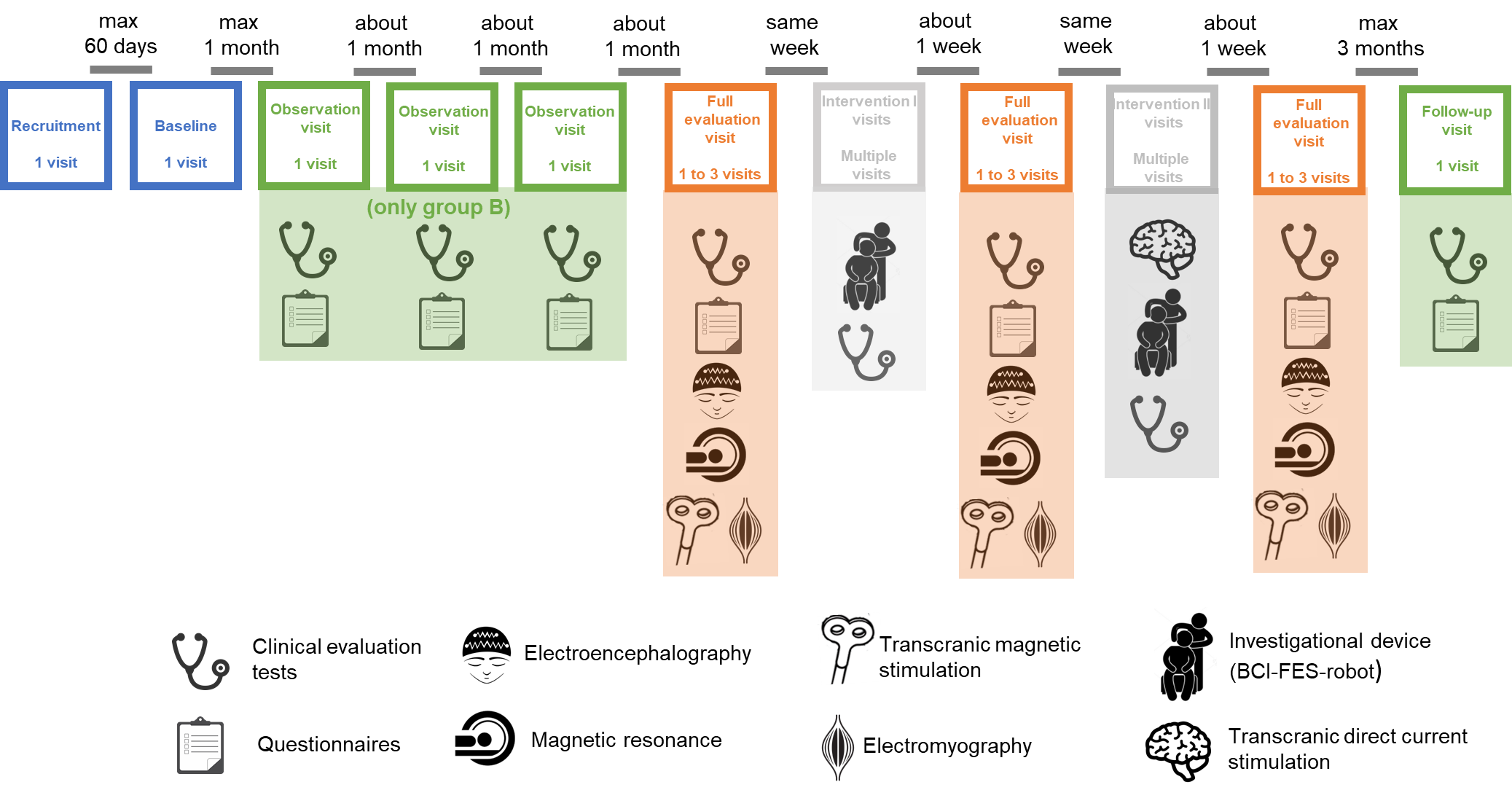


Figure 2. Scheme of planned visits and procedures.

- During each visit, a qualified physical therapist will be present, sometime (in particular during treatments) accompanied by a research engineer trained in the study.
- The **assessment visits** and the **observation visits** include the execution of clinical tests to evaluate upper limb mobility and functionality and completing questionnaires to assess your motivation and cognitive status. The participants in Group B attend 3 visits (one every month) at the beginning of the study. All participants attend a **follow-up visit** 3 months after the end of the last visit of intervention II.
- The **full evaluation visits** include the same clinical tests and additional tests, as :
  - Quality of life and motivation questionnaires,
  - Questionnaires to test the cognitive state,
  - An instrumented evaluation of the movements of the upper limbs. This includes mobility exercises, some performed during a Magnetic Resonance Imaging (MRI) examination, where participants will be lying down in the magnetic scanner and asked to rest, or to imagine themselves performing hand movements. The MRI examination may be accompanied by an electroencephalogram (EEG), during which participants will wear a cap with surface electrodes and gel to non-invasively record brain activity. During this task, they will also be asked to rest, imagine or perform hand movements.

In addition to the MRI scanner, they will also be equipped with an EEG and electromyography (EMG), which are surface electrodes that the participants will wear on their arms to measure muscle activity. During this recording, voluntary hand movements, or movements induced by transcranial magnetic stimulation (TMS), will be measured. TMS is another non-invasive and clinically accepted brain stimulation technique, and it will be performed before the brain imaging test in some evaluations. During TMS, participants will be seated in a chair and pulsed magnetic fields will be delivered by a magnet on the surface of the brain. This stimulation may or may not cause movements and can be combined with EEG and EMG measurements.

- - The quality of your sleep will be assessed for one week after the full assessment visit, with a questionnaire, a sleep diary and using a commercial device (similar to a watch) that you will wear on your unaffected arm while you sleep.

All participants attend 3 full assessment visits at the beginning, in the middle and at end of treatment.

- The number of **treatment or intervention visits** varies according to the progress you make, but they will be at least 22 (11 visits for intervention I and 11 visits for intervention II), plus calibration visits until you are able to use the BCI with at least 70% accuracy, with 2 or 3 visits per week. Rehabilitation involves the use of the investigational device to perform movements to open and close the hand and to grasp objects. Every second intervention visit, a rapid clinical evaluation test to assess your mobility progress will be performed.

Clinical tests to evaluate the mobility and functionality of the upper limb can be filmed. The questionnaires include questions about your mood, your perception of therapy and your life, and about drug and alcohol use. The time required to complete the questionnaires is approximately one hour. You are free to answer or not, especially if you find the question not appropriate.

It is possible that you can be excluded from the study before the planned term. This may occur in the following cases: (i) you ask for it; (ii) the investigator feels it is not in your best interest to continue; (iii) you do not attend at least 2 treatment visits per week for 3 consecutive weeks, or for six weeks in total since the beginning of your participation in the study; (vi) you miss assessment visits; and (v) medical complications arise, so that your eligibility criteria for the study are no longer met.

## Benefits for participants

There is no guarantee that your participation in this study will bring you any benefit. Your participation in this clinical trial allows you to practice a new long-term therapeutic rehabilitative approach that is not currently available outside a research study, and that could potentially improve the recovery of your upper limb mobility. The study may provide relevant results that can improve the rehabilitation of people who are affected by the same disease as you. If the clinical study is successful, the tested concept will inspire the design of a new device that can be used at home for upper limb rehabilitation in patients with severe chronic stroke based on the combination of the neurotechnologies proposed in this study.

## Optional nature of the participation and obligations

You are free to participate in the study or refuse to participate. You should participate in this study only on your own initiative. If you choose not to participate or if you choose to participate and reconsider your decision during the study, you will not have to justify your choice. This will not change your usual medical care. You can ask questions about the study at any time. You may decide to terminate your participation at any time. You do not have to justify your decisions. Please, contact the person indicated at the end of this information sheet.

As a study participant, you will be required to:

- Follow the investigator's instructions and comply with the study plan.
- Attend at least 2 treatment sessions per week.
- Not miss 2 sessions per week for more than 3 consecutive weeks, or for more than 6 weeks in total since the beginning of your participation in the study.
- Inform the investigator about the progress of your disease and report any new symptom, disorder, and change in your health status, and for women in childbearing age (18 to age of menopause), every month, perform a pregnancy test as proposed by the investigators.
- Inform the investigator of any concomitant treatment or therapy.
- Accept to be informed about potential accidental discoveries concerning your health.

## Risks and constraints for participants

Your participation in the study results in risk-taking related to certain examinations, to the use of the investigational device, and to the frequency of visits. Rehabilitation sessions include the use of an investigational device not yet approved for clinical use. Medical-therapy staff will be present throughout the rehabilitation period to ensure your well-being.

The risks include:

- The main risks associated with the use of the investigational device:
  - A potential excessive force of the robot on the user's hand.
  - The excessive use of electrical stimulation and robotic gloves could cause skin irritation, muscle pain, fatigue, dermatitis, tendonitis, or muscle contractures.
  - The electrical muscle stimulation is of low intensity. If a tingling sensation occurs, refer it to the investigator. He/She will then adjust the level of stimulation to avoid this discomfort. Any skin irritation or discomfort that may occur should disappear after one hour of treatment.
  - Transcranial direct current stimulation (tDCS) is a non-invasive cortical stimulation method. During tDCS, a slight tingling or itching sensation may be perceived at the site of the electrode position. We strongly encourage you to refer the experimenters if a sensation becomes unpleasant so that the stimulation can be immediately stopped.
- The frequency and duration of visits can be tiring. In this case, the rehabilitation session will be interrupted, and you will be free to stop the exercises or to continue them during the next rehabilitation session.
- Contrast-free MRI is a non-invasive technique that is widely used in the clinical field. MRI does not include exposure to x-rays but to the magnetic field. The MRI examination can be relatively noisy. For this reason, hearing protection will be provided. This also involves staying about an hour inside the scanner in a limited space. The MRI procedure is simple and safe and requires no special preparation. In order to avoid any risk related to exposure to the magnetic field, please read carefully and complete the "MRI safety" questionnaire that will be provided to you before each examination.
- TMS has been used safely in healthy participants and patients for many years for experimental and clinical purposes. Although cases of seizures have been reported under particular critical circumstances, this event is considered extremely rare and can be avoided by respecting the exclusion criteria and using a setup (intensity, duration and interval between stimuli) within the indicated safety margins, as we will do. Mild transient headaches or moderate fatigue may occur after TMS.
- The use of electrodes at each visit to record brain or muscle activity could cause slightly unpleasant sensations in the skin and it requires washing your hair at the end of the visit to remove the gel. Applying the electrodes to the arm may require the arm to be shaved.
- Depilation and the use of electrodes can irritate the skin of people with very sensitive skin. Please, inform the investigator if you have very sensitive skin, a latex allergy or if you have dermatitis.
- No data are yet available on the effects of the investigational device on the fetus and some of the planned tests are also contraindicated for pregnant women. Pregnancy is an exclusion criterion.

There may be other risks that are not yet known. In the event of the discovery of an unknown risk, the new risks will be assessed and taken into account.

## For women in childbearing age

No data are available yet on the effects of the investigational device on the fetus and some of the tests for assessment are also contraindicated for pregnant women. Pregnancy is an exclusion criterion. If you discover that you are pregnant during the study, you must immediately inform the investigator and your participation in the study will end. Before the start of the study and once a month for the duration of the study, women in childbearing age (18 to age of menopause) will be requested to do a pregnancy test and refer the outcome to the investigators.

## Alternatives

You are not obliged to participate in the study. If you decide not to take part to it, your doctor will advise you about other options for treatment. All the devices available in the study are used independently in specialized stroke rehabilitation centers. You can ask your doctor if they are available in clinical centers near you. In addition, physiotherapy, occupational therapy, and other interventions are also provided in specialized centers and by qualified private therapists.

## Results

The study provides different results:

1. individual results that concern you directly,
2. individual results discovered by chance (fortuitous discoveries),
3. the final objective results of the study as a whole.

1. The investigator will notify you during the study of any significant new findings about you. You will be informed orally and in writing; you can then decide again if you want to continue participating in the study.

2. Fortuitous findings are "concomitant outcomes", that is, results which were not explicitly sought out, but which were obtained by chance. It can be for example imaging procedures. If the MR technician or research team detects an anomaly, we will forward your images to the Radiology Department of the Geneva University Hospitals (HUG) or of the CRR SUVA Care Sion, which will inform the person in charge of this study if further follow-up is required.

The investigator will notify you during the study about any new incidental findings (e. g. MRI) that may contribute to the prevention, diagnosis, and treatment of existing or probable diseases in the future and about any new finding that may affect the benefits of the study or your safety, and therefore your consent to participate. You will be informed orally and in writing.

We draw your attention to the fact that the data collected during this study have no medical value and are in no way intended for medical diagnosis. Therefore, failure to detect anomalies does not guarantee that diseases are not occurring or will not occur.

3. The investigator can send you, at the end of the study, a summary of the overall results.

## Data confidentiality

For the purposes of the study, we will record your personal and medical data. In the event that you have already done clinical tests similar to those proposed in this study, we would be grateful if you could inform us if you wish. Knowing the results of these previous evaluations would allow us to consolidate the results obtained in this study. Only a limited number of people can access your data in not coded form, and only to perform tasks necessary for the performance of the study. Data collected for research purposes are coded at the time of collection. Coding means that all data that identifies you (such as name, date of birth, etc.) is replaced by a code, so that people who do not know this code cannot link this data to your person. The code remains permanently in the research centre.

In the case of a publication, the data are not attributable to you as a person. Your name will never appear on Internet or in a publication. Sometimes, scientific journals require the transmission of individual data (raw data). If individual data will be required to be transmitted, they will be always coded and therefore they will not allow your identification.

Your data, in coded format only, may, with the prior consent of the Ethics Commission, be used in other research projects in Switzerland and abroad by academic or non-academic research institutions, in collaboration with the study sponsor and only for scientific purposes. The data will not be used for commercial or private purposes. You may withdraw your consent to use your data at any time. If your data has been already presented in conferences or in publications, what is existing will not be destroyed, but it will not be used further.

Videos will be recorded for the evaluation of the Fugl-Meyer scale. During the procedure, your head and face will not be filmed, or, if this is not possible, the video will be blurred and coded. In the event of withdrawal of consent for the use of the data, the videos and all other data will be anonymized. If the results using your data have already been presented at conferences or published, the existing results will be retained, but not used after withdrawal of consent.

All persons involved in the study are bound by professional secrecy. All data protection directives are respected, and you have the right to access your data at any time. Your data collected in this study may subsequently be used in future research projects in Switzerland or abroad (including in Europe and the United States).

During its conduct, the study may be subject to inspections. These can be carried out by the Ethics Commission and the Swiss regulatory authority, Swissmedic. The investigator may have to disclose your personal and medical data for the purposes of these inspections. All persons are bound by professional secrecy.

It is possible that the doctor in charge of your medical follow-up may be contacted about your state of health.

## Withdrawal from study

You can withdraw from the study at any time if you wish. The medical data collected so far will be anonymized and analyzed anyway, not to compromise the value of the study as a whole. No further data will be collected after your withdrawal.

## Compensation of participants

If you participate in this study, you will not receive any compensation. You will receive a refund for the transport (and parking) costs for you and eventually for a person who will bring you to the visits. This refund will be provided you after the first three visits and sequentially every three visits made. You should tell the examinators the indicative distance you have to travel for the visits, the type of transport you will generally use, and whether you need the assistance of another person for the trip. Your participation will have no financial consequences for you or your health insurance. If you agree to participate in this study, you also waive any commercial exploitation rights (arising from patents in particular).

## Repair of damage

The Wyss Center for Bio and Neuroengineering (promoter) that initiated the study and is in charge of carrying it out is liable for any damage you may suffer in connection with the research activities. The conditions and procedures are set by law. The promoter has signed an insurance with AXA Winterthur (Policy N 14,785,335) to be able to repair the damage for which it is responsible. If you have suffered damage, please contact the investigator, or the insurance company mentioned above.

## Financing of the study

The study is fully funded by the Wyss Center for Bio and Neuroengineering (promoter).

## Contact person(s)

In case of doubt, problems, or emergencies during or after the study, you can contact one of the following contacts at any time:

Principal investigator:

Friedhelm Hummel, Ecole Polytechnique Fédérale de Lausanne [friedhelm.hummel@epfl.ch](mailto:friedhelm.hummel@epfl.ch), Tel. Sion : +41 79 890 75 57, Tel. Genève : +41 79 890 36 81.

# Declaration of consent

# Written declaration of consent for the participation in the clinical trial

Please, read this form carefully. Feel free to ask questions if you don't understand something or if you need clarifications.

| BASEC Number:  (After submission to the competent ethics committee): | 2019-00094 |
| --- | --- |
| Title of the study:  (Scientific title and usual title) | ***AV***AN***C***ER : **A**ccident **V**asculaire cérébral et **A**pport des **N**eurotechnologies individualisées chez la personne avec une hémiparésie **C**hronique : une **E**tude clinique prospective visant à **R**estaurer la mobilité du membre supérieur |
| **Responsible institution:** (Promoter with full address): | Wyss Center for Bio and Neuroengineering  Chemin des Mines, 9  1202 Genève |
| Clinical site: | □ SUVA Care, Sion  □ Campus Biotech, Genève |
| **Physician-investigator in charge of the project on site:** (Surname and first name in capital letters) : | Hummel Friedhelm |
| **Participant:**  (Surname and first name in capital letters) :  Date of birth: | ________________________________  ____ / ____ / ____ |

- I declare that I have been informed, orally and in writing, by the undersigned investigator responsible for this study about the objectives and the conduct of the study, as well as the expected effects, advantages, potential harms and potential risks.
- I am participating in this study on a voluntary basis and I accept the content of the information sheet provided to me on the above-mentioned study. I had enough time to take my decision.
- I received satisfactory answers to the questions I asked in relation to my participation in the study. I will keep the information sheet and the copy of my declaration of consent that I will receive.
- I have been informed about the existence of other treatments and therapies.
- I agree to attend at least 2 treatment visits per week, and not to miss 2 treatment sessions per week for more than 3 consecutive weeks, nor more than 6 weeks in total since I began participating in the study.
- I agree that my general doctor can be informed on my participation in the study.
- I agree that the specialists of the study sponsor, the competent Ethics Commission and the Swiss authority (Swissmedic) may consult my raw data in order to carry out controls, provided that the confidentiality of these data is strictly ensured.
- I agree to be videotaped as part of the study. My face will be blurred in any publication.
- I know that pregnancy is an exclusion criterion and that before the start of the study and once a month during the duration of the study, childbearing women will be required to do a pregnancy test.
- I am aware that my personal data may be transmitted possibly also abroad for research purposes in the context of this project only, and only in coded form.
- If I will benefit / should benefit from medical treatments outside this study, I agree that the investigator may contact the attending physicians in order to obtain relevant medical data for this study.
- I can, at any time and without having to justify myself, revoke my consent to participate in the study, without that this has an adverse effect on my further care.
- I am aware that an insurance has been subscribed to cover any damage attributable to the study that I may incur.
- I am aware that the obligations mentioned in the information sheet for participants must be respected throughout the duration of the study. The study management may exclude me at any time in the interest of my health.

| Place, date of receipt and date of signature in the presence of the investigator. | Participant’s signature |
| --- | --- |

Investigating physician’s statement: I hereby certify that I have explained to the participant the nature, importance, and scope of the study. I declare that I meet all obligations in relation to this project in accordance with the law in force. If I will become aware at any time during the course of the project, about factors that may affect the participant's consent to participate in the project, I will inform the participant immediately.

| Place, date | Surname and name of the investigating physician in capital letters.  Investigating physician signature |
| --- | --- |

**Written declaration of consent for the reuse of data in coded form (for the reuse of data from THIS research study)**

| **Participant:**  (Surname and name in capital letters):  Date of birth: |  |
| --- | --- |

I agree that my data obtained in this study may be reused for medical research purposes. This consent is valid for an unlimited period of time.

I give my consent voluntarily and I can revoke my decision at any time. If I reconsider my decision, my data will be made anonymous. I simply have to inform the investigating physician and I do not have to justify my decision.

I know that my data is stored in a coded form and that the identification list is kept in a safe place. The data may be sent for analysis to another institution located in Switzerland or abroad, provided that it complies with standards and requirements at least equivalent to Swiss standards and requirements. All legal provisions relating to data protection are respected.

Generally, the data are used in a global way and the results are published in a synthetic way. In the event that the data analysis reveals a discovery relevant to my health, the investigator will contact me.

I waive any right to commercial exploitation of my data.

| Place, date | Participant’s signature |
| --- | --- |

**Statement of the investigating doctor / person providing the information:** I hereby certify that I have explained to the participant the nature, importance, and extent of the reuse of data.

| Place, date | Surname and first name of the investigator providing information to participants in capital letters.  Signature of the investigating physician / information provider |
| --- | --- |

Full name, telephone and/or email of the general doctor.
