# Supplementary figures and images for: A Novel Patient-Tailored, Cumulative Neurotechnology-Based Therapy for Upper-Limb Rehabilitation in Severely Impaired Chronic Stroke Patients: The AVANCER Study Protocol
Source: Front Neurol. 2022 Jul 7;13:919511. doi: 10.3389/fneur.2022.919511 (PMC9301337; doi:10.3389/fneur.2022.919511)

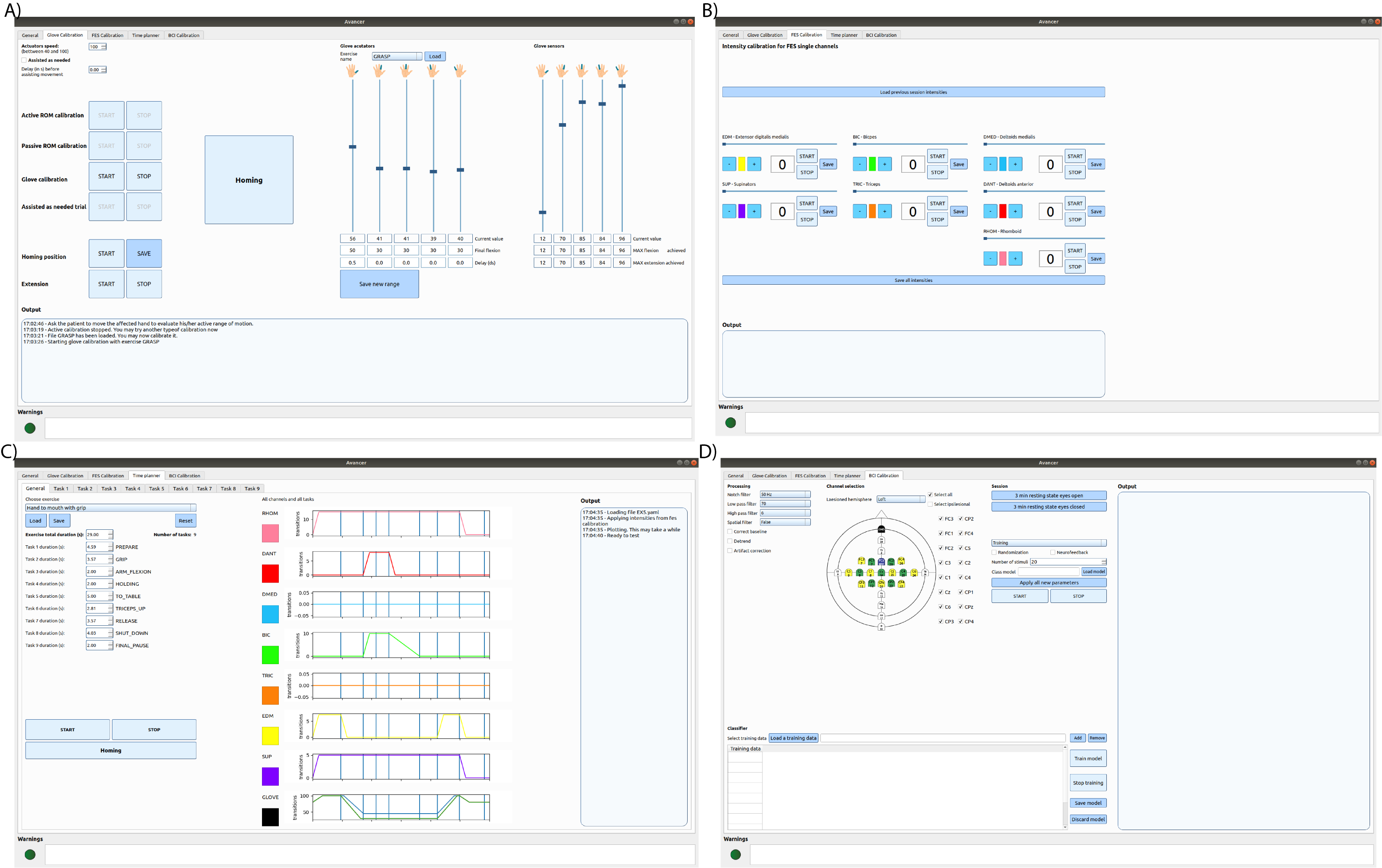

Supplement: Supplementary file 4 [file Image_1.TIF]

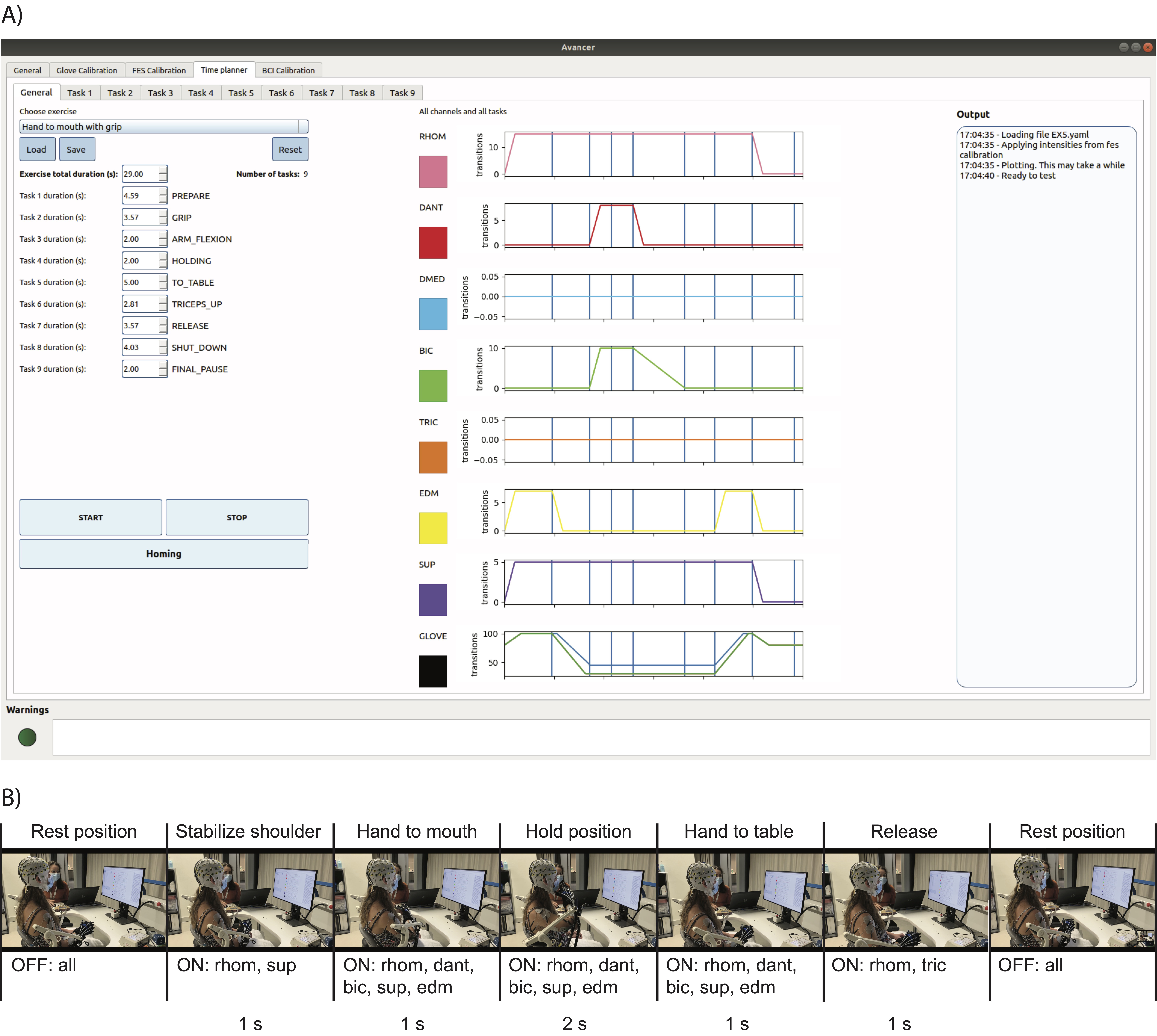

Supplement: Supplementary file 5 [file Image_2.JPEG]

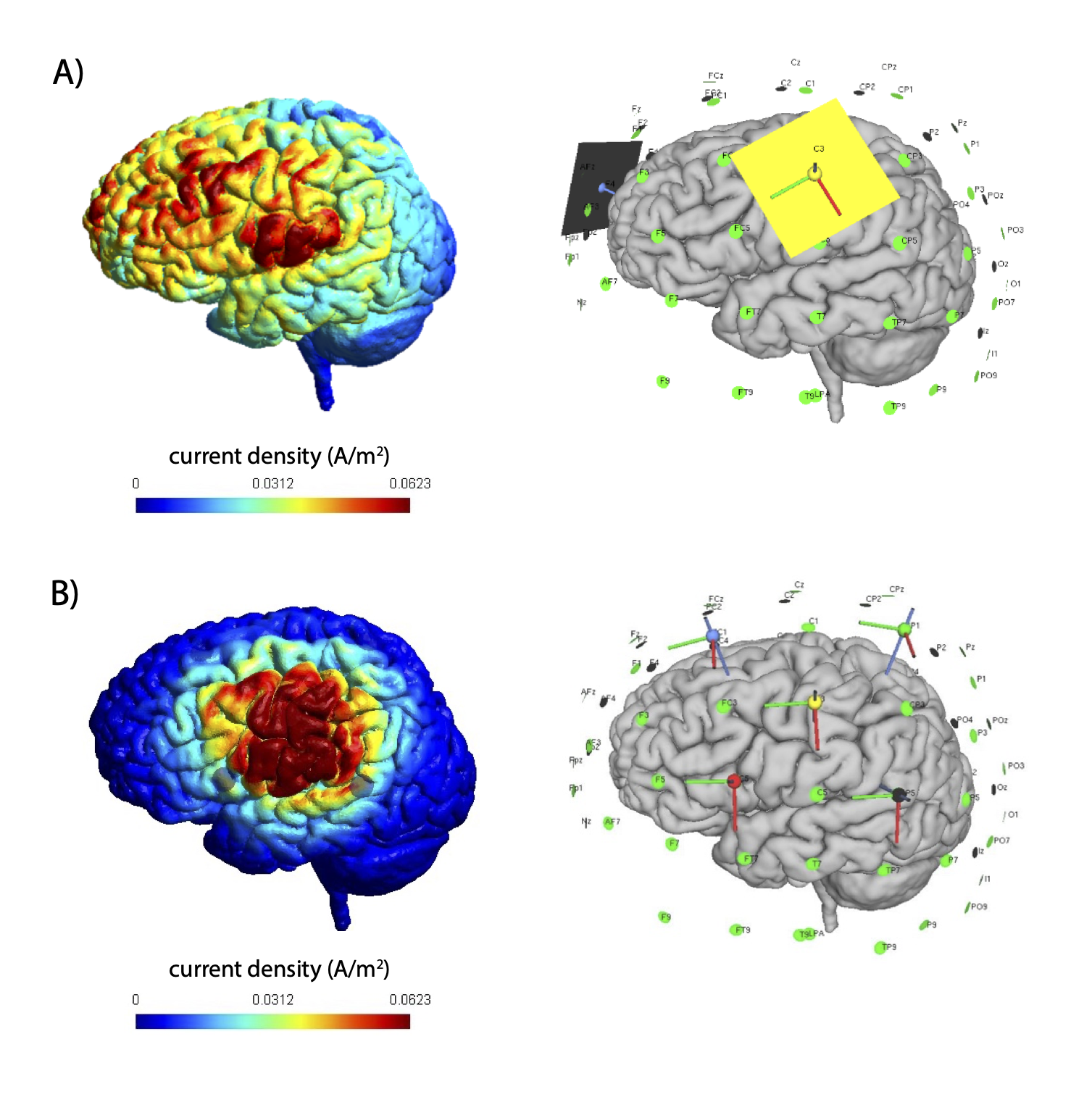

Supplement: Supplementary file 6 [file Image_3.TIFF]
